# Anthropometric measurements as a key diagnostic tool for familial partial lipodystrophy in women

**DOI:** 10.1186/s13098-024-01413-w

**Published:** 2024-09-04

**Authors:** Victor Rezende Veras, Grayce Ellen da Cruz Paiva Lima, Ivana da Ponte Melo, Virginia Oliveira Fernandes, Fabia Karine de Moura Lopes, Camila Lopes do Amaral, Maria Helane Gurgel Castelo, Larissa Luna Queiroz, Jessica Silveira Araújo, Cynthia Melissa Valerio, Renan Magalhães Montenegro Junior

**Affiliations:** 1 Brazilian Group for the Study of Inherited and Acquired Lipodystrophies (BRAZLIPO), Fortaleza, CE Brazil; 2 Clinical Research Unit, Walter Cantídio University Hospital, Federal University of Ceará/EBSERH, Fortaleza, CE Brazil; 3 Department of Clinical Medicine, Federal University of Ceará, Fortaleza, CE Brazil; 4 Department of Community Health, Federal University of Ceará, Fortaleza, CE Brazil; 5 University of Fortaleza, (UNIFOR), Fortaleza, CE Brazil; 6 IEDE, Rio de Janeiro, RJ Brazil; 7 Diagnosticos das Americas DASA, São Paulo, Brazil

**Keywords:** Familial partial lipodystrophy, Lipodystrophy, Body composition, Dual-energy X-ray absorptiometry (DXA)

## Abstract

**Background:**

Familial Partial Lipodystrophy (FPLD) is a disease with wide clinical and genetic variation, with seven different subtypes described. Until genetic testing becomes feasible in clinical practice, non-invasive tools are used to evaluate body composition in lipodystrophic patients. This study aimed to analyze the different anthropometric parameters used for screening and diagnosis of FPLD, such as thigh skinfold thickness (TS), Köb index (Köbi), leg fat percentage (LFP), fat mass ratio (FMR) and leg-to-total fat mass ratio in grams (LTR), by dual-energy X-ray absorptiometry, focusing on determining cutoff points for TS and LFP within a Brazilian population.

**Methods:**

Thirty-seven patients with FPLD and seventy-four healthy controls matched for body mass index, sex and age were studied. Data were collected through medical record review after signing informed consent. All participants had body fat distribution evaluated by skinfolds and DXA measures. Fasting blood samples were collected to evaluate glycemic and lipid profiles. Genetic studies were carried out on all patients. Two groups were categorized based on genetic testing and/or anthropometric characteristics: FPLD+ (positive genetic test) and FPLD1 (negative genetic testing, but positive clinical/anthropometric criteria for FPLD).

**Results:**

Eighteen (48.6%) patients were classified as FPLD+, and 19 (51.4%) as FPLD1. Unlike what is described in the literature, the *LMNA* variant in codon 582 was the most common. Among the main diagnostic parameters of FPLD, a statistical difference was observed between the groups for, Köbi, TS, LFP, FMR, and LTR. A cutoff point of 20 mm for TS in FPLD women was found, which is lower than the value classically described in the literature for the diagnosis of FPLD. Additionally, an LFP < 29.6% appears to be a useful tool to aid in the diagnosis of these women.

**Conclusion:**

Combining anthropometric measurements to assess body fat distribution can lead to a more accurate diagnosis of FPLD. This study suggests new cutoff points for thigh skinfold and leg fat percentage in women with suspected FPLD in Brazil. Further studies are needed to confirm these findings.

## Background

Familial Partial Lipodystrophy (FPLD) is a disease with broad clinical and genetic variation [1, 2]. Seven different subtypes of FPLD have been described. However, the genetic inheritance of FPLD type 1 (FPLD1), or Köbberling syndrome, has not yet been identified [3]. Guíllin and colleagues proposed a measure to assist in the diagnosis of patients with FPLD1, known as the Köb index (Köbi), which is calculated by the ratio of subscapular (SS) and calf skinfold (CS) thickness. According to this study, a Köbi > 3.477 is highly suggestive of this syndrome, with a sensitivity of 89% and specificity of 84% [4]. The other subtypes range from 2 to 7 and have specific genetic variants: *LMNA*,* PPARG*,* PLIN1*,* CIDEC*,* LIPE*, and *CAV1*, respectively [3–5]. The subtypes of FPLD have in common the selective loss of adipose tissue, most commonly in the lower limbs [3, 6–8].

The diagnosis of FPLD is challenging due to its clinical and phenotypic variability, with atypical and typical forms described in the literature [2, 9]. Although its diagnosis is essentially clinical, non-invasive tools are used to evaluate body composition in lipodystrophic patients [10]. These include skinfold thickness measurement, bioimpedance analysis, magnetic resonance imaging (MRI), computerized tomography scan (CT) and dual-energy X-ray absorptiometry (DXA). Among these methods, thigh skinfold thickness (TS), central-to-peripheral mass ratio (or fat mass ratio [FMR]) by DXA, and Leg-to-total fat mass ratio in grams (LTR) by DXA are commonly used for FPLD diagnosis due to their ease of use and availability (3,5,10,11). The diagnosis of FPLD can be supported by the following criteria: TS < 22 mm for women and < 10 mm for men (3,11), FMR > 1.2 [3, 11], LTR < 25% [10], and Köbi > 3.477 [4].

To calculate FMR and LTR, more in-depth knowledge of FPLD is needed since it is mandatory to identify which measurements from the DXA report should be used in each index. The leg fat percentage (LFP), a straightforward measurement, evaluates the adipose tissue proportion in the lower limbs. A recent study examining DXA parameters for diagnosing FPLD in women determined that LFP is the optimal objective anthropometric measure for diagnosis [10].

This study aimed to analyze the different anthropometric parameters used for screening and diagnosis of FPLD, focusing on determining cutoff points for TS and LFP within a Brazilian population.

## Patients and methods

### Study population

In this cross-sectional study, we identified 37 patients with previous FPLD diagnosis, clinical or genotypic, who were followed at the Endocrinology outpatient clinic of the Federal University of Ceará (Fortaleza, Brazil). This clinic is a reference in FPLD care in northeast Brazil.

The exclusion criteria were as follows: age under 18 years, male sex, acquired lipodystrophies, congenital generalized lipodystrophy (CGL), severe renal or hepatic diseases, depression and alcoholism.

Two groups were categorized based on genetic testing and/or anthropometric characteristics. FPLD + patients were those with a positive genetic variant for FPLD-related genes. Those who met clinical and anthropometric criteria for FPLD but had negative genotyping were classified as FPLD1.

FPLD1 and participants had at least three of the following: loss of adipose tissue affecting the lower limbs post-puberty, noticeable veins and muscularity (essential criteria), acanthosis nigricans, polycystic ovarian syndrome (PCOS), type 2 diabetes (T2D) or impaired fasting glucose (IFG), hypertriglyceridemia or low high-density-lipoprotein (HDL) cholesterol. The diagnostic criteria for PCOS were oligomenorrhea and hirsutism without any other known cause.

For comparison of anthropometric data, a control group with 74 healthy volunteers matched for age, sex, and body mass index (BMI) in a 2:1 ratio was selected. This group was recruited from outpatient clinics and hospital employees and was not related to the patients. This study was conducted in accordance with the ethical guidelines of the Helsinki Declaration and received approval from the Ethics Committee. Patients provided informed consent, and data were collected through medical record review after obtaining consent.

### Anthropometrical parameters

The subsequent criteria were used to support FPLD diagnosis: TS < 22 mm, FMR > 1.2, LTR < 25% and/or Köbi > 3.477.

### Laboratorial parameters

All patients underwent molecular analysis through a genetic panel for lipodystrophies and pancreatitis as an outpatient routine. The genes assessed in this panel are *ABCA1*,* AGPAT2*,* AKT2*,* APOA5*,* APOC2*,* BSCL2*,* CAV1*,* CAVIN1*,* CFTR*,* CIDEC*,* CTRC*,* CYP27A1*,* GPIHBP1*,* LIPA*,* LIPE*,* LMF1*,* LMNA*,* LMNB2*,* LPL*,* MFN2*,* PLIN1*,* POLD1*,* PPARG*,* PRSS1*,* PSMB8*,* SMPD1*,* SPINK1* and *ZMPSTE24*.

For the purpose of diagnosing FPLD, variants in the genes *LMNA*, *PPARG*, *PLIN1*, *CIDEC*, *LIPE*, and *CAV1* were taken into consideration. Adhering to the guidelines outlined by the American College of Medical Genetics and Genomics and the Association for Molecular Pathology, genetic variations in *LIPE* and *CIDEC* genes are deemed pathogenic exclusively in the homozygous state due to their autosomal recessive inheritance. Conversely, the remaining genetic variants are classified as pathogenic in both homozygous and heterozygous presentations, as their inheritance follows an autosomal dominant pattern.

The American Diabetes Association diagnostic standards for T2D and IFG were used. Dyslipidemia was diagnosed using triglycerides ≥ 150 mg/dL and/or HDL-cholesterol levels < 50 mg/dL. Fasting blood samples were collected to evaluate glycemic and lipid profiles.

### Body composition evaluation

Body evaluation by skinfolds and DXA is routinely performed by two experienced nutritionists from the clinic. Fasting weight, height, and skinfold thickness of the TS, SS and CS were measured with a calibrated Lange^®^ caliper. BMI was calculated by dividing weight (kg) by height squared (m^2^). Whole-body, truncal, upper limb, and lower limb fat mass were measured using DXA scan (GE Healthcare, model Lunar Prodigy Advance, software enCORE version 17), following the manufacturer’s recommendations for positioning, scan protocols, and analysis.

### Statistical analysis

Statistical analysis was performed using RStudio version 22.07.1 and Microsoft Excel 2016. Unpaired t tests were used for parametric variables, Kruskal-Wallis and then Mann–Whitney tests were used for nonparametric variables. A significance level of 5% was adopted. The sensitivity and specificity of TS and LFP in patients with FPLD were calculated using receiver operating characteristic curve (ROC) analysis.

## Results

Of the 37 identified patients, 18 (48.6%) were classified as FPLD + and 19 (51.4%) as FPLD1. The median age and follow-up time of the entire group were 44 and 2 years, respectively.

The anthropometric characteristics of each group are detailed in Table 1. The clinical characteristics of each participant are shown in Tables 2 and 3 of this article. When analyzing only regarding the medians of the anthropometric parameters defining lipodystrophy for all patients, we noted Köbi 4.66 (0.84-14; ± 6.33), TS 11 mm (5–55; ± 8.5), FMR 1.46 (1.07–2.16; ± 0.3), LTR 0.22 (10.3–29.3; ± 0.05), and LFP 25% (10.4–48,7; ±8,3).

**Table 1:** Comparison of anthropometric and dual-energy X-ray absorptiometry measurements between different types of familial partial lipodystrophy and healthy controls

			Groups						
Variables	N	Total^1^	Control *N* = 74^1^	FPLD+, *N* = 18^1^	FPLD1, *N* = 19^1^	*p* value^2^	Control vs. FPLD+	Control vs. FPLD1	FPLD + vs. FPLD1
Age (yo)	111	44 ± 12 (44)	43 ± 12 (43)	45 ± 15 (42)	50 ± 8 (52)	0.053	0.6	0.1	0.056
BMI (kg/m^2^)	111	27.2 ± 4.5 (26.5)	26.8 ± 4.4 (26.4)	26.1 ± 4.3 (25.3)	29.5 ± 4.7 (28.8)	**	0.8	0.056	0.068
SS (mm)	111	29 ± 11 (28)	26 ± 9 (19)	31.8 ± 15 (31)	37 ± 12 (39)	*	0.3	*	0.4
CS (mm)	63	14 ± 8 (11)	20 ± 9 (19)	3 ± 4 (6)	13 ± 6 (13)	*	*	**	*
Köbi	63	3.35 ± 2.88 (2.35)	1.49 ± 1.25 (1.12)	6.12 ± 3.4 (5.75)	3.28 ± 1.73 (3.14)	*	*	*	**
TS (mm)	111	27 ± 14 (27)	33 ± 11 (33)	8.2 ± 3 (8)	20 ± 12 (17)	*	*	*	*
WFP (%)	111	37 ± 8 (38)	40 ± 6 (40)	29 ± 8 (31)	37 ± 7 (38)	*	*	0.3	**
LFP (%)	111	35 ± 10 (35)	39 ± 7 (38)	22 ± 7 (20)	30 ± 7 (28)	*	*	*	**
TFP (%)	111	40 ± 8 (41)	41 ± 7 (41)	34 ± 9 (37)	42 ± 8 (42)	**	*	0.9	**
FMR	111	1.21 ± 0.3 (1.13)	1.06 ± 0.22 (1.02)	1.6 ± 0.32 (1.63)	1.45 ± 0.69 (1.35)	*	*	*	0.3
LTR	111	0.3 ± 0.1 (0.31)	0.98 ± 0.13 (1)	0.20 ± 0.04 (0.21)	0.22 ± 0.05 (0.23)	*	*	*	0.4

^1^ Mean ± Standard Deviation (Median); n (%). ^2^ Kruskal-Wallis Test; Fisher’s Exact Test. * *p* < 0.001. ** *p* < 0.05

Notes: BMI, body mass index; CS, calf skinfold; FMR, fat mass ratio; Köbi, Köb index; LFP, leg fat percentage; LTR, leg-to-trunk ratio; N/A, not available; SS, subscapular skinfold; TFP, trunk fat percentage; TS, thickness skinfold; yo, years old; WFP, whole fat percentage

**Table 2:** Summary of clinical and genotypic characteristics of familial partial lipodystrophy positive genotype patients

Case, age, sex	Genotype	Clinical lipoatrophy	Fat deposition	BMI	Diabetes treatment	A1c	PCOS	Comorbidities	Dyslipidemia	Higher triglycerides value (mg/dL)	Current Dyslipidemia treatment
A1, 25, F	LMNA p.(Arg582Cys)	Four limbs	DC, H	25,6	MTF (IFG)	5,2	yes	no	no	83	no
B1, 53, F	LMNA p.(Arg582Cys)	Lower limbs	DC, H, ABD	28,06	MTF, PIO, INS (2,48)	9,6	yes	HBP, mild HE, DR, DPN	↓HDL	392	ATO
B2, 58, F	LMNA p.(Arg582Cys)	Lower limbs	H, ABD	27,3	MTF, PIO, GLI^&^	13,9	menopause	HBP	↑TGL	307	no
C1, 29, F *	LMNA p.(Arg582Cys)	Four limbs	No	19,0	MTF, PIO, INS (1,83)	8,1	yes	Moderate HE, CAN	↓HDL,↑TGL	4459	ATO, CIP
C2, 31, F *†	LMNA p.(Arg582Cys), ABCA1	Four limbs	DC, ABD	20,3	MTF, PIO	6,8	no	Mild HE, DPN	↓HDL,↑TGL	1264	CIP
D1, 33, F	LMNA p.(Arg482Trp)	Four limbs	DC, ABD	23,6	MTF, PIO	8,8	yes	Mild HE, DPN	↓HDL,↑TGL	245	ATO
D3, 67, F	LMNA p.(Arg482Trp)	Four limbs	ABD	22,9	MTF, GLI	6,3	menopause	HBP, liver Tx by NAFLD, PAD, CKD, DR, DPN	↓HDL	248	SIN
E1, 52, F	LMNA p.(Arg582Cys)	Four limbs	ABD	24,5	MTF (IFG)	5,7	no	HBP, mild HE, CAD, CAN	↓HDL,↑TGL	379	ATO
F1, 33, F	LMNA p.(Arg582Cys)	Lower limbs	DC, H, ABD	32,3	INS (0,75)	8,5	yes	Mild HE	↑TGL	100	no
F2, 30, F	LMNA p.(Arg582Cys)	Lower limbs	DC, H, ABD	31,6	MTF (IFG)	5,1	yes	no	↓HDL,↑TGL	193	no
F3, 42, F	LMNA p.(Arg582Cys)	Four limbs	DC, H, ABD	30,9	No (newly diagnosed diabetes)	9,6	yes	No	↓HDL,↑TGL	185	SIN
G1, 57, F	LMNA p.(Arg582Cys)	Four limbs	DC, ABD	23,1	MTF	6,3	menopause	HBP, CI, CKD, mild HE	↓HDL,↑TGL	471	ATO
G3, 59, F	LMNA p.(Arg582Cys)	Four limbs	DC, H, ABD	28,8	MTF, INS (0,75)		menopause	no	↓HDL,↑TGL	254	SIN
H1, 32, F	LMNA p.(Arg582Cys)	Four limbs	DC, H, ABD	25,5	MTF, PIO, GLIC	8	yes	Mild HE	↓HDL,↑TGL	4523	ATO, EZE, CIP
I1, 23, F	LMNA p.(Arg582Cys)	Lower limbs	DC, H, ABD	35,2	MTF (IFG)	5,8	yes	no	↓HDL,↑TGL	254	no
J1, 41, F	PPARG p.(Leu298Profs*41)	Lower limbs	ABD	20,1	MTF, INS (2,21)	10,2	yes	DPN	↓HDL,↑TGL	816	no
L1, 38, F	PPARG p.(Leu298Profs*41)	Lower limbs	DC, H, ABD	26,0	MTF, PIO, GLI, ALO, INS (1,7)	8,9	yes	HBP	↓HDL,↑TGL	218	ROS
L2, 36, F	PPARG p.(Leu298Profs*41)	Four limbs	DC, ABD	23,5	MTF (IFG)	5,7	no	no	↓HDL,↑TGL	511	GEM

* Homozygous variant in LMNA gene

† Homozygous variant in ABCA1 gene

& Does not accept insulin therapy

# LMNA and PPARG variants are classified as pathogenic in both homozygous and heterozygous presentations, as their inheritance follows an autosomal dominant pattern, according to the guidelines of the American College of Medical Genetics and Genomics and the Association for Molecular Pathology

# The numbers in parentheses represent the amount of insulin units per kilogram of weight

Notes: ABD, abdominal; ALO, alogliptin; ATO, atorvastatin; CAD, coronary artery disease; CAN, cardiovascular autonomic neuropathy; CIP, ciprofibrate; CKD, chronic kidney disease; DC, double chin; DE, erectile dysfunction; HE, hepatic steatosis; EZE, ezetimibe; F, female; G, hump; GEM, gemfibrozil; GLI, gliclazide; HAS, systemic arterial hypertension; HDL, high density lipoprotein; IC, heart failure; INS, insulin; M, male; MMSS, upper limbs; MMII, lower limbs; MTF, metformin; NAFLD, non-alcoholic fatty liver disease; PAD, peripheral arterial obstructive disease; PIO, pioglitazone; PND, diabetic polyneuropathy; RD, diabetic retinopathy; ROS, rosuvastatin; SIN, simvastatin; TGL, triglycerides; TX, transplant

**Table 3:** Summary of clinical and genotypic characteristics of familial partial lipodystrophy type 1 (Köbberling) group

Case, age, sex	Genotype	Clinical lipoatrophy	Fat deposition	BMI	Diabetes treatment	A1c	PCOS	Comorbidities	Dyslipidemia	Higher triglycerides value (mg/dL)	Current Dyslipidemia treatment
K1, 37, F	Negative	Four limbs	DC, H, ABD	37,7	MTF, PIO, GLI, INS (1,2)	10,0	yes	HBP	↓HDL,↑TGL	382	ATO
K2, 54, F	Negative	Four limbs	H, ABD	25,9	MTF, PIO, INS (1,9)	11,8	menopause	HBP, DR, DPN	↓HDL,↑TGL	540	ROS, EZE
K3, 54, F	Negative	Four limbs	DC, ABD	28,0	MTF, INS (1,48)	9,0	menopause	HBP, mild HE, DR, CI	↓HDL,↑TGL	345	SIN, CIP
K4, 61, F	Negative	Four limbs	DC, H, ABD	30,6	MTF, INS (1,77)	7,6	menopause	HBP, DR, DPN, CAD, CI, CKD	↓HDL,↑TGL	191	ATO
K5, 44, F	Negative	Four limbs	DC, H, ABD	30,0	MTF, PIO, INS (1,55)	12,0	Yes	HBP, mild HE, DPN	↓HDL,↑TGL	9900	ROS, EZE, CIP
K6, 42, F	Negative	Four limbs	DC, ABD	22,1	MTF	4,9	No	HBP	↓HDL,↑TGL	263	SIN
K7, 55, F	Negative	Four limbs	DC, ABD	28,8	MTF	7,2	menopause	Mild HE	↓HDL	156	SIN
K8*, 52, F	AGPAT2, ABCA1	Lower limbs	DC, ABD	31,5	MTF, PIO, INS (0,7)	9,4	menopause	HBP, DPN	↓HDL,↑TGL	315	ATO
K9, 59, F	Negative	Four limbs	ABD	26,4	MTF, PIO, GLI, INS (2,09)	10,0	yes	HBP, DPN, CAD, CI	↓HDL,↑TGL	1075	ATO, CIP
K10, 40, F	Negative	Lower limbs	DC, H, ABD	26,0	MTF, INS (1,1)	11,9	menopause	DPN	↓HDL,↑TGL	5745	ATO, CIP
K11, 55, F	Negative	Lower limbs	DC, H, ABD	39,7	MTF, INS (1,88)	9,0	No	HBP, DR, DPN, CI	↓HDL,↑TGL	172	ATO, CIP
K12, 29, F	Negative	Upper limbs	DC, H, ABD	39,9	MTF (IFG)	6,1	menopause	HBP	↓HDL,↑TGL	191	no
K13, 42, F	Negative	Lower limbs	DC, H, ABD	26,2	MTF, INS (2,04)	9,2	yes	DPN, CAD, CI	↓HDL,↑TGL	1869	ATO, EZE, CIP
K14, 62, F	Negative	Four limbs	DC, H, ABD	30,9	MTF, PIO, DAP, INS (1,0)	6,7	No	HBP, DPN, CAN, CAD, CI	↓HDL,↑TGL	336	ATO
K15, 56, F	Negative	Lower limbs	DC, ABD	21,6	MTF, GLI^&^	9,6	menopause	DPN, CAD	↓HDL,↑TGL	266	CIP
K16, 49, F	Negative	Four limbs	DC, ABD	28,8	MTF, INS (0,84)	8,0	menopause	HBP, DR, CAD, CKD	↓HDL,↑TGL	334	ROS, EZE
K17, 39, F	Negative	Four limbs	DC, H, ABD	33,2	MTF, INS (1,85)	6,5	No	HBP, DR, DPN	↓HDL,↑TGL	162	ATO
K18, 40, F **	LPL p.Glu448Lys	no	DC, H, ABD	26,7	MTF, INS (1,2)	7,1	No	HBP, DPN	↓HDL,↑TGL	321	ATO
K19, 60, F **	LPL p.Asn318Ser	Upper limbs	DC, H, ABD	35,5	MTF, EMP, INS (1,28)	9,7	menopause	HBP, DR, DPN, CKD	↓HDL,↑TGL	1872	ATO

* Heterozygous variant in the AGPAT2 and ABCA1 genes

** Heterozygous variant in the LPL genes

# Heterozygous variants in LPL, AGPAT2 and ABCA1 genes are classified as variant of uncertain significance, according to the guidelines of the American College of Medical Genetics and Genomics and the Association for Molecular Pathology

# The numbers in parentheses represent the amount of insulin units per kilogram of weight

Notes: ABD, abdominal; ATO, atorvastatin; CAD, coronary artery disease; CI, cardiac insufficiency; CIP, ciprofibrate; CKD, chronic kidney disease; DC, double chin; DPN, diabetic polyneuropathy; DR, diabetic retinopathy; EZE, ezetimibe; F, female; H, hump; GLI, gliclazide; HBP, high blood pressure; HDL, high density lipoprotein; HE, hepatic steatosis; INS, insulin; M, male; MTF, metformin; NAFLD, non-alcoholic fatty liver disease; PAD, peripheral artery disease; PIO, pioglitazone; ROS, rosuvastatin; SIN, simvastatin; TGL, triglycerides

The FPLD + group (*N* = 18) includes individuals from 11 different families, all of whom are native to the state of Ceará. The average follow-up time was 5.2 years, with a median age of 45 years. Three patients reported familial consanguinity, and one was an adopted daughter. Fifteen patients (83.3%) had Dunnigan Syndrome (FPLD2), belonging to nine different families. The mean age was 40.6 years, and the mean follow-up time was 5.3 years. Among FPLD2 patients, twelve (80%) had *LMNA* p.(Arg582Cys) variant, while three (20%) harbored the *LMNA* p.(Arg482Trp) variant. Two sisters (Cases C1 and C2, Table 2), born to consanguineous parents, had homozygous *LMNA* p.(Arg582Cys) variant, presenting with the generalized phenotype. These two cases were previously reported in the literature [12]. Three women belonging to two lineages carried *PPARG* p.(Leu298Profs*41) variant. It was not possible to analyze FPLD + subgroups separately due to the small sample of patients with *PPARG* gene variants. In this group, only two women carrying the LMNA p.(Arg582Cys) variant did not satisfy the FMR criteria (Cases C1 and G3, Table 2); notably, one of these individuals presented the variant in a homozygous state. Both TS and FMR were frequently identified as diagnostic criteria in the FPLD + group, with all FPLD + patients meeting the criteria when TS was employed.

Seventeen (89.5%) of the FPLD1 group had obesity or overweight. FMR was ≥ 1.2 in all individuals of the group. Eight FPLD1 patients had a Köbi > 3.477, of whom seven had TS < 22 mm, and only one patient, with a BMI of 37.6 kg/m2, had a TS of 23.5 mm. The remaining patients in the group did not show positivity for Köbi. The median BMI for all groups remained within the overweight range (p 0.04). The leanest individuals belonged to the FPLD + group.

A significant *p* value was observed in the analysis between all groups for Köbi, TS, LFP, FMR, and LTR (*p* < 0.001). When comparing controls versus FPLD + and controls versus FPLD1 for the same parameters mentioned above, *p* < 0.001 was found to all comparisons.

When correlating the diagnostic criteria among FPLD groups, we found statistically significant differences in the comparison for Köbi (p 0.016). However, when analyzing the components of this index, there was a significant difference between these two groups only for CS (*p* < 0.001). Regarding TS, the FPLD + group was different from FPLD1, with a *p* value of < 0.001 for both comparisons. The parameters that showed similarity between FPLD subtypes were LTR and FMR.

**Fig. 1:**
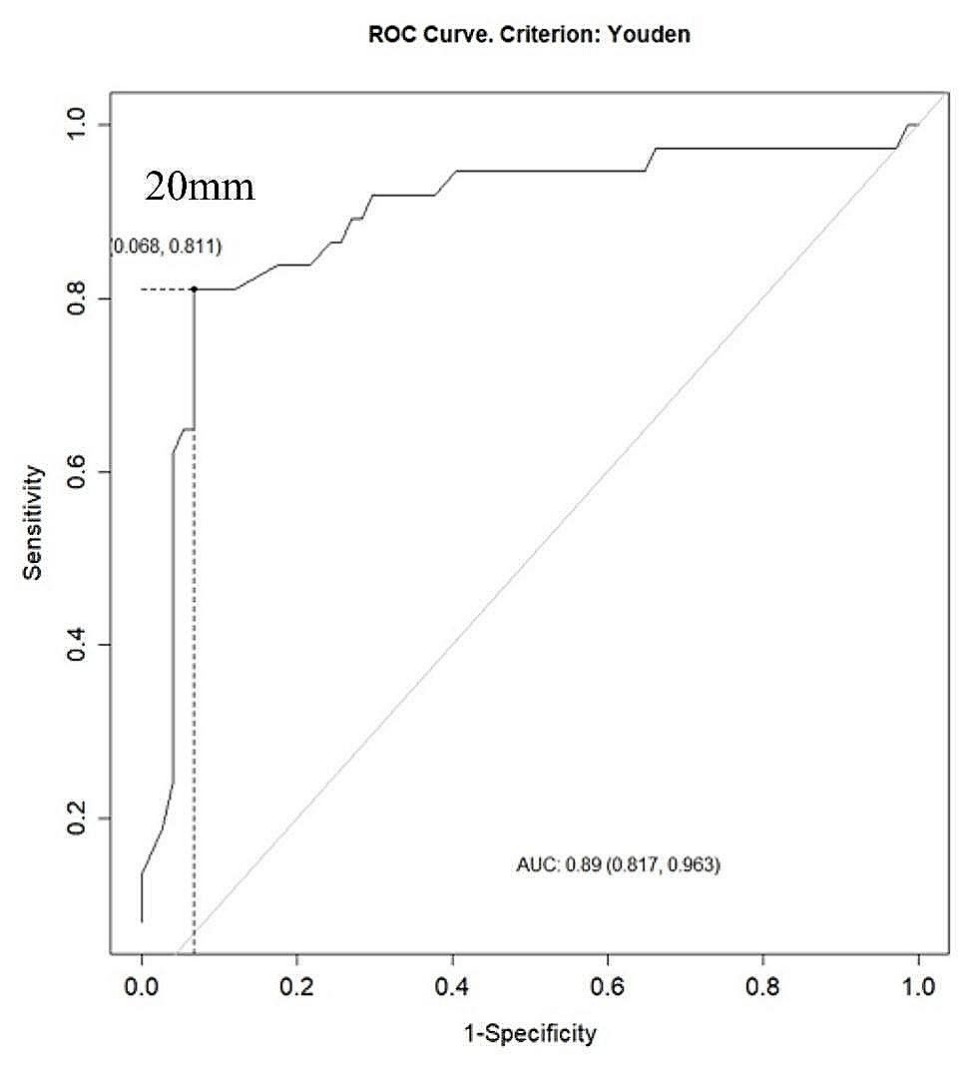
ROC curve for thigh skinfold thickness cutoff point in Familial Partial Lipodystrophy women by Youden’s criterion

**Fig. 2:**
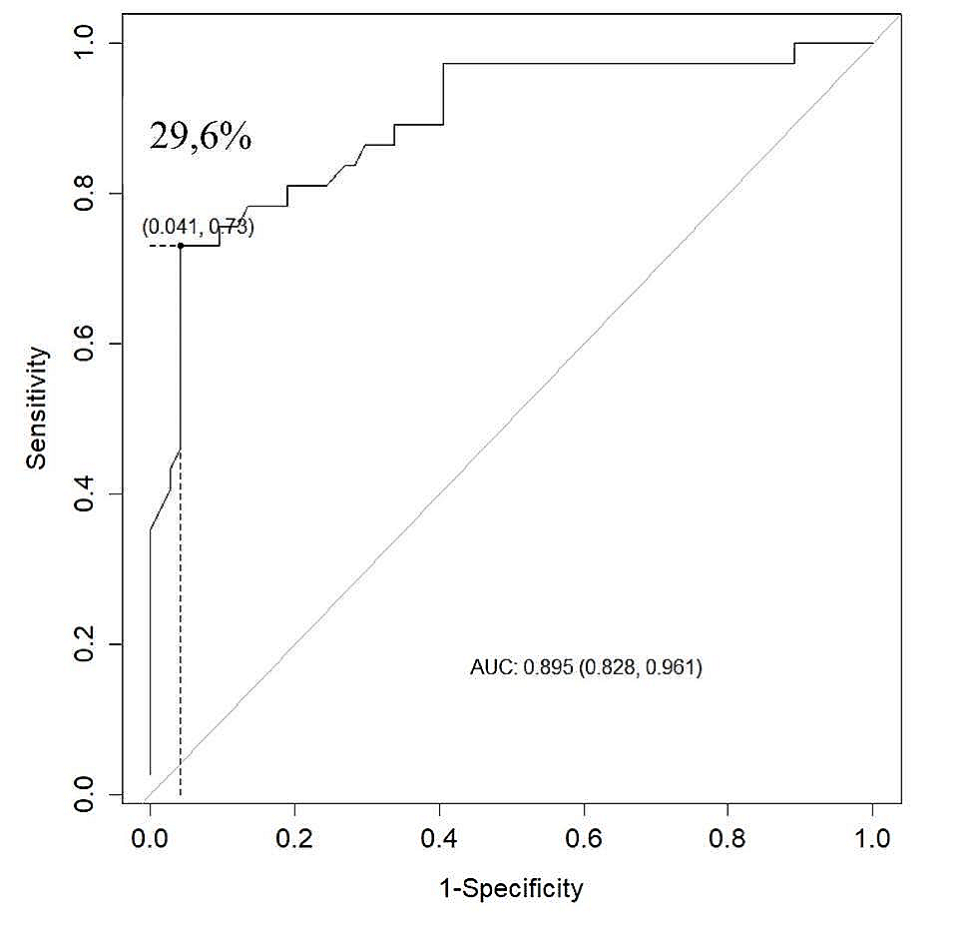
ROC curve for leg fat percentage cutoff point in Familial Partial Lipodystrophy women by Youden’s criterion

Lipoatrophy of the lower limbs is a prominent feature observed during the evaluation of FPLD. TS represents one of the main parameters for the diagnostic support of lipodystrophic syndromes. Based on its relevance in the literature, we analyzed a cutoff point of TS in the sample. The cutoff point by Youden’s criterion with the best balance was 20 mm, with a sensitivity of 81%, specificity of 93.2%, and area under the curve (AUC) of 0.89 (Fig. 1). Furthermore, LFP cutoff point was also evaluated as a diagnostic tool for FPLD and it was 29.6% (sensitivity 72.9%, specificity of 95.9% and AUC 0.895) (Fig. 2). For this particular group of women, the median age, TS, and LFP were 42 years, 11 mm, and 25%, respectively.

## Discussion

This is the first Brazilian study comparing different subtypes of FPLD among themselves and with a healthy control group, having evaluated 37 patients and 74 healthy controls.

It is known that genetic tests are costly and have low availability in our setting. Therefore, anthropometric analyses remain the most commonly used to support the diagnosis of lipodystrophic syndromes, possibly due to their greater simplicity of execution and lower cost, especially the performance of skinfold thickness measurements. It is important to note that FMR, LTR, and skinfold thickness measurements are indirect measures and may not be specific to FPLD and may be altered in other conditions that affect body fat distribution, such as hypercortisolism and exogenous obesity [1, 13–15]. Delayed diagnosis may contribute to increased comorbidities and complications in this population [5, 16, 17].

When analyzing the diagnostic criteria for lipodystrophies, lower limbs lipoatrophy is present, even indirectly, in several evaluative methods, such as TS, the Köbi index, and ratios between masses and fat percentages in DXA (FMR and LTR) [10, 11]. Additionally, this is a remarkable phenotypic change that captures the attention of healthcare professionals examining patients with suspected FPLD. There was a statistically significant difference in the comparison between FPLD + and FPLD1 for Köb (*p* = 0.016). However, when analyzing the components of this index, there was a significant difference for these two groups only for CS, which had a *p* value < 0.001, whereas the same was not observed in the comparison between the SS skinfolds of the groups. This finding supports the significance of lower limbs lipoatrophy in distinguishing between subtypes of FPLD lacking established genetic bases.

Diagnostic aid tools that rely on physical characteristics, such as TS, may be influenced by ethnicity, and the same cutoff points may not be applicable to different populations worldwide. TS is one of the most widely accepted and used parameters for the diagnosis of FPLD, probably due to its relative simplicity in execution. However, it requires a properly calibrated and scientifically validated skinfold caliper, as well as a professional who knows how to perform the technique properly. In addition, it is an operator-dependent test, and there may be discrepancies in measurements even among experienced examiners. These points may explain the statistically significant difference found between the groups.

The LFP may be an alternative to TS for diagnostic suspicion in scenarios where genetic testing is not feasible and the examiner lacks familiarity with other anthropometric indices of DXA, such as FMR and LTR. Other advantages of LFP include the possibility of objective documentation, easy evaluation without the need for patient privacy concerns during the consultation, low cost and quick execution compared to MRI, and low radiation dose compared to CT scan.

The available data in the current literature on TS are largely derived from non-Brazilian populations and subsequently extrapolated for use in the evaluation of the Brazilian population [4, 8]. Therefore, the cutoff values found in FPLD women for TS and LFP in this study may represent more suitable parameters for the evaluation of female patients with lipodystrophy from Brazil.

Limitations of this research include the retrospective data collection, some of which were self-reported; the small sample size, although it should be noted that this is a rare disease; the exclusively northeastern and Ceará cohort, which may limit the generalizability of the findings to other regions of Brazil; and the exclusion of the male and pediatric population from the study.

## Conclusion

The combined use of anthropometric measurements for assessing body fat distribution, clinical history, and, if possible, genetic analysis contributes to a definitive and more accurate diagnosis of FPLD. A new cutoff point for thigh skinfold and leg fat percentage in women in this case series was suggested, which are 20 mm and 29,6%, respectively. These parameters might be deemed more suitable for assessing suspected FPLD women in Brazil. Further studies are needed to confirm these associations.

## Data Availability

All data generated or analysed during this study are included in this published article [and its supplementary information files].

## Abbreviations

AUC: Area under the curve
BMI: Body mass index
CGL: Congenital generalized lipodystrophy
CS: Calf skinfold
CT: Computerized tomography scan
DXA: Dual-energy X-ray absorptiometry
FMR: Fat mass ratio
FPLD: Familial Partial Lipodystrophy
FPLD+: Patients were those with a positive genetic variant for FPLD
FPLD1: Patients with negative genetic testing, but who met clinical and anthropometric criteria for FPLD
HDL: High-density-lipoprotein
IFG: Impaired fasting glucose
Köbi: Köb index
LFP: Leg fat percentage
LTR: Leg-to-total fat mass ratio in grams
MRI: Magnetic resonance imaging
PCOS: Polycystic ovarian syndrome
SS: Subscapular
T2D: Type 2 diabetes
TS: Thigh skinfold thickness
